# Reduction of bowel loop motion during radiotherapy for gynaecological cancer assessed by 3D cine-MRI

**DOI:** 10.1016/j.ctro.2025.101075

**Published:** 2025-11-10

**Authors:** J.J. Laan, D.L.J. Barten, Z. van Kesteren, L.J.S. Ewals, A. Bel, K.A. Hinnen, B.R. Pieters, L.J.A. Stalpers, H. Westerveld

**Affiliations:** a Amsterdam UMC Location University of Amsterdam, Department of Radiation Oncology, Meibergdreef 9, 1105 AZ Amsterdam, the Netherlands; b Erasmus MC Cancer Institute, Erasmus University Medical Center, Department of RadiationOncology, Rotterdam, the Netherlands; c Cancer Center Amsterdam, Imaging and Biomarkers, Amsterdam, the Netherlands

**Keywords:** Bowel loop motion, 3D cine-MRI, Deformable image registration, Gynecological cancer, Bowel toxicity

## Abstract

**Introduction:**

Gastrointestinal toxicity is a major concern after radiotherapy for gynaecologic cancer. Strong dose–effect relationships for the bowel and individual loops are lacking, which might be explained by challenges in accurate radiation dose estimation due to continuous motion of the bowel loops. The objective of this study was to evaluate whether changes in bowel loop motion occur over the course of radiotherapy, with the use of three-dimensional (3D) cine-MRI.

**Methods and Materials:**

This study prospectively enrolled patients with gynaecologic cancer undergoing primary radiotherapy with curative intent. To measure bowel loop motion, three 3D cine-MRI scans were acquired: before external beam radiotherapy (EBRT), in the last week of EBRT, and on the day of brachytherapy. A MR image was acquired every 3.7 s during a 10-minute scan. Deformable image registration was used to generate a motion map and a motion volume histogram. Changes in bowel loop motion over the course of treatment were assessed by the Wilcoxon signed-rank test using the median bowel loop motion in millimetres per 3.7 s of 50% of all voxels.

**Results:**

For 15 of the 22 patients, 3D cine-MR scans were available at all three time points. Median bowel loop motion per 3.7 s was 1.8 mm (range 0.6–3.3 mm) before EBRT, 1.0 mm (range 0.6–2.3 mm) in the last week of EBRT, and 0.7 mm (range 0.4–1.1 mm) at brachytherapy. Most patients (14 out of 15) showed reduced bowel loop motion in the last week of EBRT compared to the first MR, with the lowest motion at brachytherapy in 13 patients (87 %). Median bowel loop motion decreased significantly with each subsequent MRI scan (*p* = 0.005 and *p* = 0.020, respectively).

**Conclusions:**

The results of this study demonstrate a statistically significant decrease in bowel loop motion over the course of primary radiotherapy for gynaecological cancer.

## Highlights

• Bowel loop motion decreases during EBRT for gynaecological cancer.
• The lowest bowel loop motion was observed at the time of brachytherapy.
• Six of 22 patients had prior surgery, but no difference in baseline bowel loop motion was found.

## Graphical abstract

**Figure:**
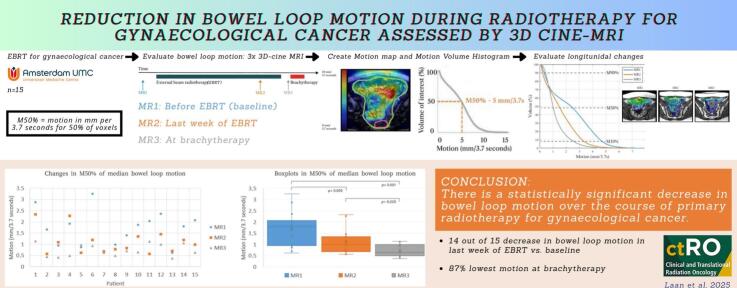

## 1 Introduction

Each year, approximately 5000 women are treated for gynaecological cancer in the Netherlands [1]. Primary pelvic radiotherapy with or without concurrent chemotherapy usually followed by a brachytherapy boost is the standard of care for patients with locally advanced, inoperable, disease. One-third of patients treated with pelvic radiation experience a moderate to severe decrease in quality of life due to radiation-induced gastrointestinal toxicity, defined as pelvic radiation disease [2]. Between 6–12 % of patients undergoing primary chemoradiation for cervical cancer experience chronic, severe gastrointestinal complications as a result of the treatment, including ulcerations and fistulae [[3], [4], [5]].

Late bowel toxicity is typically attributed to the radiation dose delivered. Late bowel toxicity was found to correlate more closely with the dose received by specific bowel loops than by the entire bowel bag in a study of 135 cervical cancer patients [6]. A systematic review of dose-volume predictors for late bowel toxicity indicate that there is currently insufficient evidence to provide dose constraint recommendations for external beam radiotherapy (EBRT) for the large bowel, and bowel loops [7]. Furthermore, the Quantitative Analyses of Normal Tissue Effects in the Clinic (QUANTEC) only reports bowel loop volume constraints for acute toxicity and does not report a maximum dose or constraints for late bowel toxicity [8]. One potential explanation for the observed lack of dose-volume predictors for late bowel toxicity is the motion of bowel loops [9,10]. Individual loops demonstrate a range of motility patterns and are capable of altering their position within the peritoneal cavity, as well as segmentation (by contraction of circular muscles) and peristalsis (the contraction of longitudinal muscles that causes the bowel to move in a wave-like motion). A change in the position of the bowel loops may result in an underestimation or overestimation of the dose received in comparison with the projected dose based on the planning CT or MRI. This is particularly relevant in the context of brachytherapy, where a relatively high dose with steep dose gradients is delivered over a comparatively short period of time in close proximity to the bowel loops. Furthermore, there may be an increased risk of bowel toxicity in patients with bowel loops fixed by adhesions as a result of previous abdominal surgery in the proximity of the high-dose region [3,11].

The Groupe Européen de Curiethérapie and the European Society for Radiotherapy & Oncology (GEC-ESTRO) recommend the implementation of 3D MRI guidance in the treatment of cervical cancer [12]. This approach includes the use of MRI prior to EBRT and at the time of brachytherapy. In a previous study, our research group developed a novel three-dimensional (3D) cine-MRI acquisition technique that allows quantification of bowel loop motion and identification of regions with high or low bowel loop motion [13]. By extending the standard of care MRI by 7.5 min for the cine-MRI, it became possible to identify bowel loop motion variations as small as 0.5 mm [14].

The purpose of this study was to use 3D cine-MRI to evaluate whether changes in bowel loop motion occur during radiotherapy for gynaecological cancer, including both the change during EBRT and at brachytherapy.

## 2 Material and Methods

This prospective study, the “BOMOPI” study, was approved by the Institutional Medical Ethics Committee and registered in the Central Committee for Research on Human Subjects (CCMO) database under number NL65554.018.18. It included patients with histologically confirmed gynaecological cancer, including those with FIGO stage I-IVA cervical or vaginal cancer and isolated vaginal recurrence of endometrial cancer. All patients were treated with primary radiotherapy with curative intent, either with or without concurrent chemotherapy or hyperthermia. The treatment regimen consisted of EBRT with 25 fractions of 1.8 Gy to the pelvic region, administered in five fractions per week, followed by a brachytherapy boost in one or two applications. All patients underwent brachytherapy application under general anaesthesia. Postoperatively, patient-controlled epidural analgesia (PCEA) was the standard of care. Alternatively, patients received an opioid patient-controlled analgesia (PCA) pump. All enrolled patients gave written informed consent. Exclusion criteria included any contraindication to MRI, such as claustrophobia, metallic foreign bodies, or a cardiac implantable electronic device.

The objective was to enrol at least 10 evaluable patients who all received at least 3 three-dimensional (3D) cine-MRIs, and including at least three patients with a history of major abdominal surgery. In the final analysis, 22 patients were included in the study, of whom 15 underwent three evaluable cine-MRIs. The bowel loop motion was evaluated using 3D cine-MR imaging at three time points. Imaging was conducted prior to the start of radiotherapy (baseline), in the last week of EBRT (pre brachytherapy) and at the time of brachytherapy with the applicator in place (at brachytherapy). At each of these three time points, a clinical MRI was part of the standard of care in accordance with the GEC-ESTRO guidelines for MRI-guided adaptive brachytherapy [12]. Scans were acquired with a 3 Tesla Philips Ingenia MRI scanner (Philips, Best, The Netherlands). The cine-MRI was acquired prior to the administration of the spasmolytic agent Scopolamine (BUSCOPAN, Boehringer, Ingelheim, Germany) required for the clinical protocol. Patients were not given any oral intake instructions prior to the MRI at baseline and in the last week of radiotherapy. At brachytherapy (MR3), the majority of patients were in a state of fasting, having recently undergone a surgical procedure including general analgesia. Bowel loop motion was assessed using a 10-minute scan with 3D images acquired every 3.7 s. This resulted in an MRI movie with 160 3D images. Voxel-wise displacements between consecutive images were estimated using deformable registration with a B-spline transformation model implemented in the Elastix toolbox (version 4.900) [15]. The algorithm optimized a mutual information similarity measure, with registrations performed over the entire image (rather than just within the area of interest). The B-spline grid spacing was 2.5 mm, the gray-level histogram was sampled with 32 bins, and optimization was run for 1000 iterations. Given the large number of deformable image registration operations, automated quality assurance was required. For each deformation vector field (DVF), we calculated the Jacobian determinant and Harmonic Energy to assess whether the transformations were biomechanically plausible, physiologically realistic, and numerically robust[[16], [17], [18]]. For motion analysis, two metrics were defined, the maximum and median bowel loop motion. To quantify the maximum displacements during the 10-minute acquisition, the 95th percentile maximum displacement per voxel was calculated across 159 DVFs. Each DVF consisted of 160 × 160 × 50 voxels, with a voxel size of 1.25 × 1.25 × 2.5 mm^3^. Each DVF described the magnitude and direction of motion between consecutive dynamics (3.7 s). Given that the 3D cine-MRI comprised 160 dynamics, a total of 159 DVFs were generated per acquisition.

This metric, previously termed VL95 in our earlier study, is now referred to as ‘maximum bowel loop motion’ and is used to evaluate the impact of major abdominal surgery on baseline bowel loop motion [14]. Regions exhibiting low maximum motion demonstrate persistent low motion over the course of the 10-minute scanning period. One potential cause of this persistent low motion is fixated bowel loops resulting from intra-abdominal adhesions. All other results and analyses were based on the 50th percentile displacement per voxel from the same 159 vector fields, now referred to as ‘median bowel loop motion’. This metric was previously defined as VL50 in prior studies. The median bowel loop motion shows the most frequent motion in the region of interest during the 10-minute scan acquisition time. A robust evaluation of changes in bowel loop motion over time and between patients is achieved through the use of median bowel loop motion. Both metrics of bowel loop motion were used to construct motion maps. Further clarification of the methodology and accuracy of the 3D cine-MRI and motion metrics used in this study can be found in our previous publications [13,14].

To minimize the potential for identifying regions of low motion (e.g., bone) or high motion (e.g., respiratory motion of the abdominal muscles) other than the bowel loops, the volume of interest was defined as the bowel bag, excluding the bladder, uterus, and rectum. The bowel bag, defined as the bowel region between L5 and the symphysis, was manually delineated on only the first of 160 dynamics of each 3D cine-MRI using Velocity (Velocity 4.1, Varian Medical Systems, Inc., of Palo Alto, California, United States). The bladder, cervix, uterus, and rectum were delineated and subsequently subtracted from the delineated bowel bag.

**Fig. 1:**
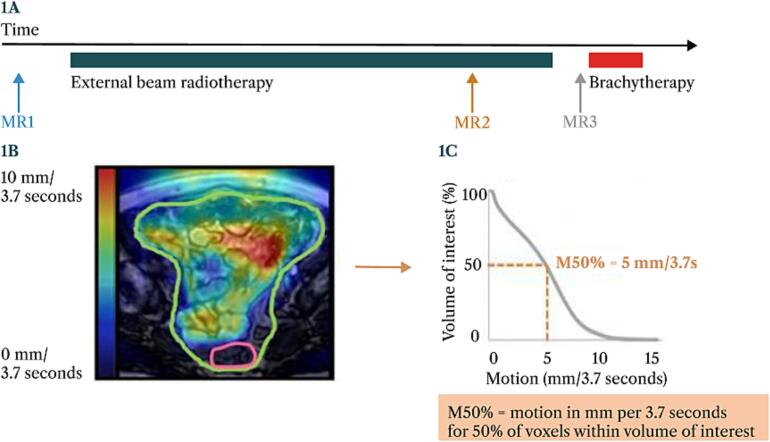
*Schematic representation illustrating the timing of the three cine-MRIs (1A) and an example of a cine-MRI acquired motion map (1B) and its corresponding motion volume histogram (1C).***1A**. MR1, baseline; MR2, last week of external beam radiotherapy; MR3, at brachytherapy. **1B.** Motion map of median bowel loop motion. Red: regions with high median motion. Blue: regions with low median motion. Green delineation: bowel bag. Pink delineation: rectum. 1C. Motion Volume Histogram (MVH) of median bowel loop motion. X-axis: median motion in the volume of interest (bowel bag minus rectum and uterus). Y-axis: percentage of that volume that has motion equal to or greater than the x-value. (For interpretation of the references to colour in this figure legend, the reader is referred to the web version of this article.)

For both motion metrics (maximum and median bowel loop motion), motion-volume histograms (MVHs) were generated from the motion maps and the volume of interest to assess changes in bowel loop motion throughout the course of treatment. The analysis focused on three previously established MVH parameters: M10%, M50%, and M90%[13]. The M50% metric was used to assess differences in bowel loop motion between and among individual patients. The M50% is defined as the minimum motion per 3D image (per 3.7 s) in 50 % of the voxels within the volume of interest. In the example shown in Fig. 1C, the median bowel loop motion is at least 5 mm per 3.7 s for 50 % of the volume within the volume of interest (=M50%). The M10% is defined as the minimum motion per 3D image in 10 % of the voxel, and the M90% is defined as the minimum motion per 3D image in 90 % of the voxels. In the motion map, regions with high median motion are indicated in red, while regions with low median motion are indicated in blue. Fig. 1 illustrates a schematic representation of the timing of 3D cine-MRIs and an example of an axial slice of the bowel loop motion map acquired by 3D cine-MRI. Regions of high or low motion were not specifically considered during treatment planning.

Patient and treatment characteristics were obtained from the medical records. All types of surgery associated with the risk of intra-abdominal adhesions were considered major abdominal surgery, including recent oncologic surgery such as lymphadenectomy or radical hysterectomy [3]. Simple hysterectomy was also considered major abdominal surgery. The absence of the uterus after hysterectomy allows the bowel loops to be located close to the top of the vagina. The use of medication during radiotherapy that may affect bowel loop motion, including laxatives, loperamide, opioids, and antiemetics, was also extracted from the medical records. Only systematic use of these medications as prescribed for at least three consecutive days during EBRT was evaluated. The use of these medications in an incidental manner was not included in the scoring. The 22 patients were divided into two groups based on their baseline bowel loop motion, with the median value serving as the cutoff point. Severe acute toxicity was scored according to the CTCAE version 5.0, defined as grade three or higher, occurring within three months during or after radiotherapy [19]. Baseline patient and treatment characteristics were also scored separately for patients with high and low baseline bowel loop motion (with the median bowel loop motion as a cut-off). The Mann-Whitney *U* test was used to assess the differences in baseline M50% of the maximum bowel loop motion between patients with and without a history of major abdominal surgery. In the 15 patients who underwent all three 3D cine-MRIs, the changes in M50% of median bowel loop motion throughout the course of treatment were analysed. The potential impact of concurrent chemotherapy on the absolute change in the M50% of median bowel loop motion during EBRT (MR2 minus MR1) was evaluated using the Mann-Whitney *U* test. In addition, the Mann-Whitney *U* test was used to assess differences in the absolute change in M50% of median bowel loop motion between the last week of radiotherapy and brachytherapy (MR3 minus MR2) for patients who received epidural anaesthesia versus those who received other postoperative pain control. Spearman's rank correlation was used to assess the correlation between alterations in weight during EBRT and changes in bowel loop motion observed during EBRT (MR2 minus MR1). It should be noted that there is potential for variability in the field of view of each MRI, with differences observed between scans of the same patient. Consequently, only the overlapping parts within the volume of interest of all MRIs (based on automated rigid registration around the spinal vertebrae) were compared using the Wilcoxon signed-rank test. The data were analysed using SPSS version 28.0.1.1 (IBM Corp. Released 2019. Armonk, NY, USA).

## 3 Results

### 3.1 Patient and treatment characteristics

Patients were enrolled in this study from June 2018 until September 2019. During this period, 48 patients received EBRT with curative intent for gynaecological cancer at our centre. Twelve patients did not consent to participate in the study. Fourteen patients were excluded from the study due to not meeting the inclusion criteria. Of these, one patient with a body mass index (BMI) of 56 kg/m^2^ was excluded from the analysis afterwards due to poor image quality of the MRI and two patients had contraindications for MRI, specifically a hip prosthesis and an MRI incompatible metal clip. In the final analysis, 22 patients were included with at least one baseline 3D cine-MRI, and 15 patients were included with all three available MRI’s.

Table 1 presents the baseline patient and treatment characteristics of the entire cohort. Although conclusions should be drawn cautiously, patients with high bowel motion at baseline seemed to use loperamide more often during EBRT.

**Table 1:** Baseline patient and treatment characteristics for the entire cohort and divided into two groups based on the median bowel loop motion at baseline.

Variable		Entire cohort		Low bowel loop motion		High bowel loop motion	
		n = 22	(%) or IQR	n = 11	(%) or IQR	n = 11	(%) or IQR
Age^a^	Median in years and IQR	57	45–66	52	35–64	65	50–71
WHO Performance score	0	19	(86 %)	10	(91 %)	9	(82 %)
	≥1	3	(14 %)	1	(9 %)	2	(18 %)
Body Mass Index^a^	Median and IQR	26.5	24–30	26.5	(25–30)	25.7	(24–30)
Smoking	Current smoker	3	14 %	3	(27 %)	0	(0 %)
Diabetes Mellitus	n (%)	1	4 %	0	(0 %)	1	(9 %)
Major abdominal surgery^a^	n (%)	8	(36 %)	2	(18 %)	6	(55 %)
*(of which) hysterectomy*	*n*	*5*		*1*		*4*	
Primary tumor *Cervical cancer*	*n (%)*	*17*	*(77 %)*	*9*	*(82 %)*	*8*	*73 %)*
*Vaginal cancer*	*n (%)*	*3*	*(14 %)*	*1*	*(9 %)*	*2*	*(18 %)*
*Endometrial cancer*	*n (%)*	*2*	*(9 %)*	*1*	*(9 %)*	*1*	*(9 %)*
Nodal status	N0	17	(77 %)	10	(91 %)	7	(64 %)
	N1	5	(23 %)	1	(9 %)	4	(36 %)
Medication during radiotherapy	n (%)						
*Opioid*	*n (%)*	*4*	*(18 %)*	*2*	*(18 %)*	*2*	*(18 %)*
*Antiemetic*	*n (%)*	*6*	*(27 %)*	*3*	*(27 %)*	*3*	*(27 %)*
*Loperamide*	*n (%)*	*10*	*(45 %)*	*3*	*(27 %)*	*7*	*(64 %)*
*Laxative*	n (%)	*4*	*(18 %)*	*1*	*(9 %)*	*3*	*(27 %)*
Brachytherapy fractions	2 fractions	13	(59 %)	7	(64 %)	6	(55 %)
	1 fraction	9	(41 %)	4	(36 %)	5	(46 %)
EBRT extended-field (PAO)	n (%)	1	(4 %)	0	(0 %)	1	(9 %)
SIB lymph nodes	n (%)	5	(23 %)	2	(18 %)	3	(27 %)
EBRT dosimetric parameters							
*V30 bowelbag*	*Median and IQR (cc)*		*280 (62–420)*		*228 (57–541)*		*281 (154–397)*
*V40 bowelbag*	*Median and IQR (cc)*		*154 (35–263)*		*155 (27–294)*		*153 (73–222)*
Concurrent chemotherapy	n (%)	12	(54 %)	5	(55 %)	7	(64 %)
Concurrent hyperthermia	n (%)	6	(27 %)	3	(27 %)	3	(27 %)

IQR; Interquartile range, WHO; World Health Organization, EBRT; External Beam Radiotherapy, PAO; *para*-aortal, SIB; simultaneous integrated boost, V30 volume of the bowelbag receiving at least 30 Gray (Gy) of radiation dose during external beam radiotherapy, V40 volume of the bowelbag receiving at least 40 Gray (Gy) of radiation dose during external beam radiotherapy,.

Low bowel loop motion, below the median at baseline [1.7 mm/3.7 s]; High bowel loop motion, above the median at baseline.

a All types of surgery associated with the risk of intra-abdominal adhesions.

### 3.2 Toxicity and weight loss

Two patients experienced severe acute toxicity: one developed a symptomatic pulmonary embolism shortly before the brachytherapy application, and the other developed urosepsis due to pancytopenia.

Weight loss during EBRT was observed in 13 patients, with a median of 3.1 kg of these 13 patients at the end of EBRT compared to baseline (range −1.5 to −7 kg). Four patients maintained a stable weight throughout treatment. Five patients gained weight during EBRT (range +0.2 to +4.2 kg).

### 3.3 Bowel loop motion

**Fig. 2:**
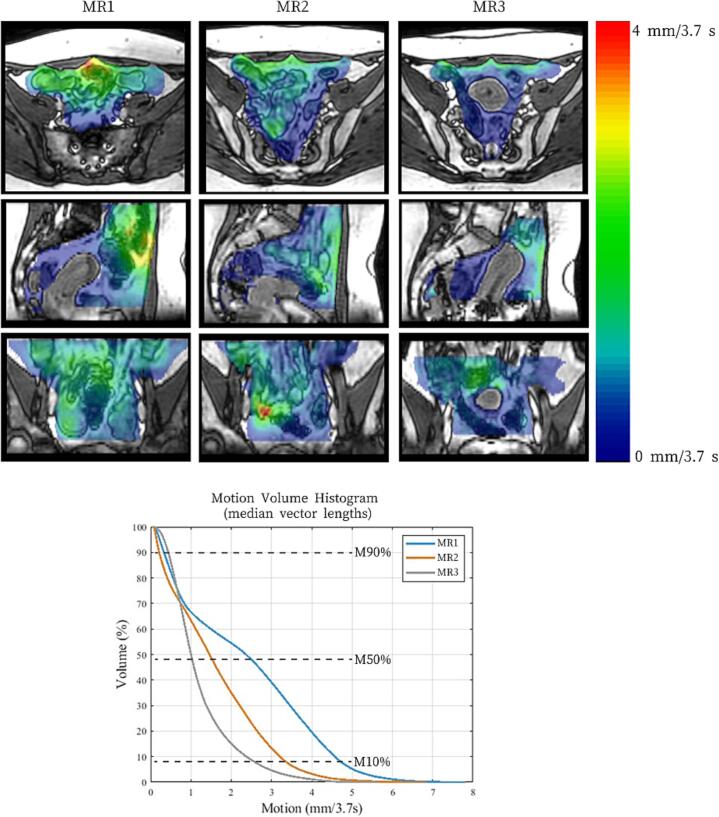
*Example of a motion map showing median bowel loop motion and corresponding motion volume histogram over the course of treatment for one patient in axial, sagittal and coronal plane*. MR1, baseline; MR2, last week of external beam radiotherapy; and MR3, at brachytherapy. Red, high bowel loop motion; blue, low motion in blue. The intersection of the coloured lines and the dotted lines in the motion volume histogram indicates various potential cut-off values for the median motion of a certain percentage of voxels within the volume of interest. Patient 8 exhibits a decrease in bowel loop motion over time. (For interpretation of the references to colour in this figure legend, the reader is referred to the web version of this article.)

**Fig. 3:**
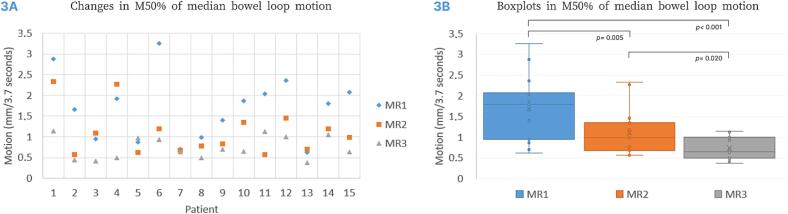
The M50% of median bowel loop motion of the 15 patients with three 3D cine-MRIs. M50%; median motion for 50 % of all voxels within the volume of interest, MR1; before external beam radiotherapy (EBRT) in blue, MR2; in the last week of EBRT in orange, MR3; at brachytherapy in grey. 3A Scatterplot showing the M50% of median bowel loop motion for all three scans for each patient. 3B Box plots comparing bowel loop motion between scans. The boxplot shows the median vector length, interquartile range, range and outliers. Comparison between M50% of median bowel loop motion of consecutive MRIs was analysed using the Wilcoxon signed-rank test. (For interpretation of the references to colour in this figure legend, the reader is referred to the web version of this article.)

Fig. 2 shows an example of axial, sagittal and coronal plane of the median motion maps and corresponding MVHs over the course of treatment for one patient (patient 8, see Fig. 3A).

Table 2 presents the MVH parameters M10%, M50%, and M90%, and the changes in median bowel loop motion between consecutive scans. The median bowel loop motion decreased over the course of treatment, with a M50% of 1.8 mm/3.7 s (range 0.6–3.3 mm/3.7 s) at baseline and 1.0 mm/3.7 s (range 0.6–2.3 mm/3.7 s) in last week of EBRT, shown in Fig. 3. In 93 % of patients (14/15) M50% decreased in the last week of EBRT compared to before EBRT. In 13/15 patients (87 %), M50% was lowest at brachytherapy (MR3) with a median of 0.7 mm/3.7 s (range 0.4–1.1 mm/3.7 s).

**Table 2:** Median bowel loop motion for the cohort of 15 patients for all three MRIs in M10 %, M50% and M90%.

MVH	**MR1** Baseline		**MR2** Last week of EBRT		**MR3** At brachytherapy		**ΔMR1 vs. MR2**		**ΔMR2 vs. MR3**	
	Median motion	(range)	Median motion	(range)	Median motion	(range)	Median motion	(range)	Median motion	(range)
M10% in mm/3.7 s	3.6	(1.6–6.9)	2.8	(1.2–4.6)	1.8	(0.8–3.9)	−0.6	(−5.5 to + 1.1)	−0.8	(−2.7 to + 2.6)
M50% in mm/3.7 s	1.8	(0.6–3.3)	1.0	(0.6–2.3)	0.7	(0.4–1.1)	−0.6	(−2.1 to + 0.1)	−0.3	(−1.2 to + 0.6)
M90% in mm/3.7	0.3	(0.2–0.8)	0.2	(0.2–0.8)	0.2	(0.2–0.5)	0.0	(−0.3 to + 0.1)	0.0	(−0.6 to + 0.2)

vs.; versus, M10 %, M50% and M90 % is defined as the motion in mm per 3.7 s (each 3.7 s an image is acquired) for respectively 10 %, 50 % and 90 % of all voxels within the volume of interest.

Legends: Δ or delta; change in median bowel loop motion between two scans, ΔMR1 vs. MR2 shows the difference in motion in mm/3.7 s between these scans calculated as MR2 minus MR1. ΔMR 2 vs. MR3 shows the difference in median motion in mm/3.7 s between these scans calculated as MR3 minus MR2.

Fig. 3A shows the M50% of the median bowel loop motion for all 15 patients with three 3D cine-MRIs. Fig. 3B shows the box plots of the M50% of median bowel loop motion of all overlapping area of interest of MR1, MR2 and MR3. Bowel loop motion decreased between each consecutive MR scan. Significant differences were observed between MR1 and MR2 (*p* = 0.005), MR2 and MR3 (*p* = 0.020).

In the entire cohort of 22 patients, 6 patients had an increased risk of abdominal adhesions due to a medical history of previous major abdominal surgery. However, M50% of maximum bowel movement at baseline was not significantly different between patients with and without previous major abdominal surgery, 5.9 mm/3.7 s and 4.8 mm/3.7 s respectively (p = 0.238).

No significant differences were found in the complete case analysis of 15 patients evaluating the absolute change in M50% of median bowel loop motion for patients treated with and without concurrent chemotherapy (MR2 minus MR1) and for patients with postoperative epidural anaesthesia (11/15) versus other pain control after brachytherapy (MR3 minus MR2).

No significant correlation was found between alterations in weight during EBRT and change in bowel loop motion during EBRT (MR2 minus MR1) (*p* = 0.581).

**Fig. 4:**
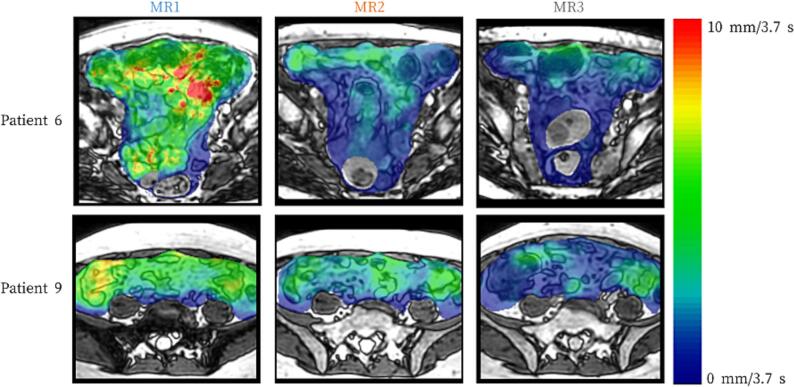
*Example motion maps of two study patients showing median bowel loop motion in M50% on the axial plane at three time points*. MR1: baseline; MR2: last week of external beam radiotherapy; MR3: during brachytherapy. Red indicates high bowel loop motion, and blue indicates low motion. Patient 6 shows higher baseline bowel loop motion than Patient 9 and a decrease over time, while Patient 9 demonstrates more stable bowel loop motion throughout. (For interpretation of the references to colour in this figure legend, the reader is referred to the web version of this article.)

Fig. 4 depicts three axial slices of each three-dimensional cine-MRI at the three time points during treatment for two patients. In patient 6, who had a higher M50% of median baseline bowel loop motion compared to patient 9, the M50% of median bowel loop motion decreased markedly both at the last week of radiotherapy and at brachytherapy. In contrast, patient 9 showed a smaller reduction in M50% of median bowel loop motion during treatment.

## 4 Discussion

This study is the first to demonstrate a decrease in bowel loop motion during primary radiotherapy for gynaecological cancer. In our cohort, there was a significant decrease of median bowel loop motion in the last week of EBRT compared to before EBRT and at brachytherapy compared to the last week of EBRT.

It remains a challenge to accurately predict severe late bowel toxicity, as a subset of patients who ultimately develop severe gastrointestinal symptoms lack any of the established risk factors. The findings of this study illustrate the considerable interpatient variability in median bowel loop motion. This could eventually allow for patient stratification, whereby other risk factors can be considered alongside bowel loop motion for a more adequate risk prediction of late severe bowel toxicity. The present study is limited by a small sample size and a limited follow-up period. Consequently, the results do not include toxicity rates or a multivariate analysis of factors that might explain or influence bowel loop motion.

In all but two patients, the lowest median bowel loop motion was observed at the time of brachytherapy, suggestion that bowel loop motion decreases during treatment.

The clinical relevance of this reduction remains uncertain and warrants further investigation.

Several mechanisms may contribute to the observed decrease. In addition to a possible effect of the radiotherapy, treatment related factors such as dietary changes due to radiation- or chemotherapy-induced mucositis should be considered, particularly as the majority of the patients included in our study were fasting at the time of brachytherapy and approximately half of the patients received concurrent cisplatin [[20], [21], [22]]. Another factor might be medication use such as loperamide, which was used more frequently in patients with a high median baseline bowel loop motion [23]. Possibly, patients with high baseline bowel loop motion were more prone to developing diarrhoea during treatment. Another observation was that a higher proportion of patients with high baseline median bowel loop motion had undergone prior major abdominal surgery. One possible explanation is that hysterectomy was classified as a major abdominal surgery in our analysis, although the associated risk of adhesions may be limited.

Larger prospective trials are needed to evaluate the relationship between bowel loop motion, treatment-related toxicity such as diarrhoea, and the potential modifying effects of medication use and surgical history.

The reported motion metrics describing the maximum and median motion in millimetres per 3.7 s can accurately determine short time-scale displacements such as peristalsis and passing of gas. However, it is not yet possible to extrapolate our metrics to the slower drifts and tracking of individual bowel loops towards or away from the high-dose region over the duration of a radiotherapy fraction, which lasts several minutes. This is needed for proper dose accumulation and of value to generate accurate dose–effect relationships for the small bowel. Moreover, some bladder filling may have occurred during the 10-minute acquisition time while we defined the area of interest for the initial dynamic scan only. This could potentially influence the MVH near the lowest motion (0 mm on the x-axis), as it would lead to fewer voxels being available for deformable image registration in the relatively small volume where the bladder filling has occurred, but it has a negligible impact on the M50%. Although a motion map can illustrate regions with increased or decreased motion, it is not feasible to compare motion maps. Our metrics, however, do allow for longitudinal comparisons, and MVH's can visualize the motion over the duration of the scan. It is therefore important to emphasize that the combination of a motion map and MVH provides a comprehensive overview in each individual patient.

The current practice is designed to maximize the sparing of the bowel and rectum. However, clinical goals are not individualized, and the current planning aims are not specific for more motile parts of the bowel. The displacement of bowel loops is of primary importance for treatment planning, in contrast to peristalsis or contraction of bowel loops, where loops ultimately remain in a similar position. The absence of identifiable anatomical landmarks in individual bowel loops prevents the tracking of each loop over time in each MRI. A decrease in median motion does not necessarily mean less planning uncertainty but perhaps more bowel loops fixated near the high dose region. A future focus would be the evaluation of bowel loop motion during brachytherapy. Movement of a bowel loop toward the high-dose region would then be observed and could be taken into account during brachytherapy treatment planning.

The previously developed 3D cine-MRI technique enables the straightforward tracking of alterations in bowel loop motion throughout the course of radiotherapy. An extension of several minutes from the standard MR imaging time allows for visualization of regions exhibiting high and low motion**.** The comparatively small number of participants (n = 22) in the present study prevented a more detailed stratification based on patient characteristics. In this hypothesis generating study, no significant differences in bowel loop motion were observed between patients with and without a history of major abdominal surgery. The next step is to investigate how the observed reduction in bowel loop motion translates to the movement of individual bowel loops during a radiotherapy fraction and its potential impact on treatment planning and toxicity. In addition, it is important to evaluate in future studies whether the radiation dose delivered to the bowel loops influences the extent of motion reduction.

Our hypothesis is that in the future, patients at risk of severe toxicity could be identified based on their motion map in combination with other known risk factors. This could, for example, reveal the presence of adhesions that immobilize bowel loops near high dose regions. In such cases, MRI- or CT-guided adaptive radiotherapy with real-time cine imaging and gated EBRT delivery, as previously demonstrated in other patient groups, could be a viable approach [24].

## 5 Conclusions

In patients with gynaecologic malignancies, median bowel loop motion decreased significantly during EBRT and at time of brachytherapy.

## Data availability statement for this work

The research data is confidential.

## CRediT authorship contribution statement

**J.J. Laan:** Conceptualization, Methodology, Investigation, Writing – original draft. **D.L.J. Barten:** Conceptualization, Methodology, Software, Visualization, Writing – review & editing. **Z. van Kesteren:** Conceptualization, Methodology, Software, Visualization, Writing – review & editing. **L.J.S. Ewals:** Data curation, Resources. **A. Bel:** Conceptualization, Writing – review & editing. **K.A. Hinnen:** Resources, Writing – review & editing. **B.R. Pieters:** Methodology, Resources, Writing – review & editing. **L.J.A. Stalpers:** Resources, Writing – review & editing. **H. Westerveld:** Conceptualization, Methodology, Supervision, Writing – review & editing.

## Funding

None.

## Declaration of competing interest

The authors declare the following financial interests/personal relationships which may be considered as potential competing interests: The authors declare no conflict of interest for the reported work. Outside of this work, author B.R. Pieters has received grants from ELEKTA, Stockholm, Sweden (payment to the institution) and a speaker/lecture honorarium from BD (to the institution), author Z. van Kesteren has received funding from General Electronics, Varian Healthcare, and Philips Healthcare (all to the institution).
